# Editorial: Complementary/Alternative Therapies for Epilepsy and Related Psychiatric Comorbidities

**DOI:** 10.3389/fnins.2022.947644

**Published:** 2022-06-09

**Authors:** Zhenghao Xu, Yi Wang, Aubin Moutal

**Affiliations:** ^1^College of Basic Medical Science, Zhejiang Chinese Medical University, Hangzhou, China; ^2^Key Laboratory of Neuropharmacology and Translational Medicine of Zhejiang, School of Pharmaceutical Sciences, Zhejiang Chinese Medical University, Hangzhou, China; ^3^Department of Pharmacology and Physiology, School of Medicine, St. Louis University, St. Louis, MO, United States

**Keywords:** epilepsy, alternative therapeutic approach, complementary and alternative medicine, psychiatric comorbidities of epilepsy, herbal medicine, anti-epileptic drugs (AEDs), stem cell therapy

Epilepsy and related psychiatric comorbidities are major public health problems affecting many millions of people across the globe. Complementary and alternative medicine (CAM) has increasingly become a viable option for treating epilepsy and related psychiatric conditions. Due to this, the advanced understanding of CAMs for epilepsy is particularly expected and valuable.

The special topic, entitled “*Complementary/Alternative Therapies for Epilepsy and Related Psychiatric Comorbidities*,” was initiated on June 4th 2020, with a dedicated team of handling editors to facilitate the timely peer-review and publication of relevant manuscripts. Within about 1 year and 9 months (from June 4th 2020 to February 28th 2022), 15 manuscripts were submitted, of which six were published, including two reviews and four original research contributions from 44 prominent scientists in the field. The content of each of these articles is summarized below.

A Hungarian study by Girgis et al. explored the use of CAM among patients with epilepsy and diabetes mellitus using a self-developed questionnaire. They found the ratio of CAM use was 9.7% in the overall study population (127 patients with epilepsy and 100 patients with diabetes mellitus). Among patients with epilepsy, the ratio of CAM use was less than that among diabetic patients; 7.9 and 12%, respectively. Of note, the most commonly used CAM here are capable of modulating cytochrome P450 enzymes and hence may result in drug interactions and adverse drug reactions. Thus, Girgis et al. further suggest that educating patients and including a clinical pharmacist specialized in the CAM field in their treatment advisory team is essential.

*Synedrella nodiflora* is a weed that grows abundantly in Ghana mainly in water-logged and shady areas. Extracts of *Synedrella nodiflora* (SNE) are used traditionally for the management of epilepsy in the West African sub-region, including Ghana. Amoateng et al. from Ghana focused on the neuronal basis of anticonvulsant effects of SNE. They found SNE not only concentration-dependently depressed excitatory synaptic transmission but also suppressed *in vitro* seizure activities induced by zero Mg^2+^ and high K^+^ -containing artificial cerebrospinal fluid in the CA1 region of the hippocampus. This preliminary study provides cellular evidence in support of the use of *Synedrella nodiflora* in traditional practice to manage convulsions.

Chinese herbal medicine has a long history of use for treating epilepsy. A review from Lin and Hsieh summarized the clinical use and mechanisms, which indicate different herbs can be combined to increase antiepileptic effects through various mechanisms, including anti-inflammation, antioxidation, enhancement of GABAergic inhibition, modulation of NMDA channels and sodium channel, and neuroprotection. Besides, they also provide evidence for the efficacy of medical diet therapy. Of note, adequate experimental evidence and randomized controlled clinical trials are still required to confirm the antiepileptic effects of most Chinese herbal medicine.

Tetramethylpyrazine (TMP) is the main activity compound of Chinese herbal medicine *Ligusticum chuanxiong Hort*. A study by Jiang et al. showed that the early application of TMP, but not in the late period, alleviates neuropsychiatric comorbidities (anxiety and depression) and increases the hippocampal BDNF/ERK expression in two different neurological diseases mouse model, including 6 Hz corneal rapid kindling model of epilepsy and Complete Freund's adjuvant (CFA) model of chronic pain. This preclinical experimental study emphasizes the importance of early treatment of psychiatric comorbidities in both epilepsy and chronic pain.

The retrospective study by Xiao et al. analyzed routinely acquired functional magnetic resonance imaging (fMRI) data of adults with refractory focal epilepsy who had clinical language fMRI scans as part of their pre-surgical evaluation at the National Hospital for Neurology and Neurosurgery (NHNN), London, United Kingdom, between January 2010 and January 2017. By comparing the effects of different drug loads of anti-seizure medications (ASM) on the verbal fluency fMRI in patients, they mainly found: (1) ASMs are associated with altered cognitive fMRI activation patterns during verbal fluency, especially for drugs with severe cognitive side effects (*n* = 119 for both moderate and severe ASMs group); (2) ASMs with moderate cognitive side effects act greater effect on fMRI activations when given in polytherapy. Thus, this study highlights the cognitive effects of adding ASMs and a high drug load of ASM. Similar to western ASMs, these phenomena may also exist in other CAMs for epilepsy, which needs to be further studied.

Except for these above drug related studies, a review of Sadanandan et al. explores the safety, efficacy, and potential mechanisms of stem cell therapy as a novel treatment for neurological and neuropsychiatric symptoms of epilepsy. Based on current preclinical findings and some clinical trials, stem cell therapy seems to benefit epilepsy and related psychiatric comorbidities. The potential mechanisms mainly include ameliorating aberrant neuronal circuitry, restoring deplete GABAergic inhibitory neurons, releasing neurotrophic factors, maintaining adenosine homeostasis, and exerting neuroprotective effects. Further preclinical investigations are still warranted to test the safety and efficacy of stem cell therapy in clinically relevant models of epilepsy; and the optimal stem cell dosage, delivery method, and timing of transplantation remain unclear.

Overall, this special topic provides an insight into the role of CAMs on epilepsy and related psychiatric comorbidities. These studies may be beneficial both in understanding how different pathophysiological processes contribute to epilepsy (and related psychiatric comorbidities) and in offering new treatment options. Of note, caution needs to be emphasized as some of the published manuscripts may have different methods of analysis, different procedures for presenting their data, or differences in interpretation of certain observations among the many different perspectives. We hope that our special topic provides useful information that will inspire other researchers to further this exciting research area.

## Author Contributions

All authors listed have made a substantial, direct, and intellectual contribution to the work and approved it for publication.

## Conflict of Interest

The authors declare that the research was conducted in the absence of any commercial or financial relationships that could be construed as a potential conflict of interest.

## Publisher's Note

All claims expressed in this article are solely those of the authors and do not necessarily represent those of their affiliated organizations, or those of the publisher, the editors and the reviewers. Any product that may be evaluated in this article, or claim that may be made by its manufacturer, is not guaranteed or endorsed by the publisher.

